# In Vitro Scolicidal Activity of the Sesquiterpenes Isofuranodiene, α-Bisabolol and Farnesol on *Echinococcus granulosus* Protoscoleces

**DOI:** 10.3390/molecules25163593

**Published:** 2020-08-07

**Authors:** Mohammad Reza Youssefi, Ali Nikpay, Niloufar Hassanpour, Aida Mirzapour, Parisa Saleh Tabari, Roman Pavela, Filippo Maggi, Riccardo Petrelli

**Affiliations:** 1 Department of Veterinary Parasitology, Babol-Branch, Islamic Azad University, Babol 19585/466, Iran; youssefi929@hotmail.com; 2 Faculty of Veterinary Medicine, Amol University of Special Modern Technologies, Amol 4618649767, Iran; ali.nikpay@gmail.com; 3 Faculty of Veterinary Medicine, Babol-Branch, Islamic Azad University, Babol 19585/466, Iran; niloofar.hasanpoour@gmail.com (N.H.); mediline_50@yahoo.com (A.M.); salehtabari9372@gmail.com (P.S.T.); 4 Crop Research Institute, Drnovska 507, Ruzyne, 161 06 Prague 6, Czech Republic; pavela@vurv.cz; 5 School of Pharmacy, University of Camerino, via Sant’Agostino 1, 62032 Camerino, Italy; riccardo.petrelli@unicam.it

**Keywords:** isofuranodiene, α-*b*isabolol, farnesol, cystic echinococcosis, protoscolex

## Abstract

Cystic echinococcosis (CE) remains an important challenge both in humans and animals. There is no safe and suitable remedy for CE, so the discovery of new compounds with promising scolicidal effects, particularly from herbal sources, is of great importance for therapeutic uses in the treatment and prevention of CE reappearance. Sesquiterpenes are C15 organic compounds made up of three isoprene units and mostly occurring as fragrant components of essential oils. They are of economic importance for the cosmetic and pharmaceutical industry, and recently attracted the attention of the scientific community for their remarkable parasiticidal properties. In the present study, we have focused on three known sesquiterpenes, isofuranodiene (IFD), α-bisabolol (BSB), and farnesol (FOH), as important phytoconstituents of the essential oils of wild celery (*Smyrnium olusatrum*), chamomile (*Matricaria chamomilla*), and acacia farnese (*Vachellia farnesiana*), respectively. Protoscoleces were recovered from fertile hydatid cysts and were exposed to different concentrations of the three tested compounds for different exposure times. The viability of protoscoleces was confirmed by 0.1% eosin staining. Results of scolicidal activity evaluations showed that IFD possessed the best effect against *Echinococcus granulosus* protoscoleces (LC_50_ and LC_90_ values of 8.87 and 25.48 µg/mL, respectively), followed by BSB (LC_50_ of 103.2 µg/mL) and FOH (LC_50_ of 113.68 µg/mL). The overall toxicity of IFD differed significantly from those of FOH and BSB, while there was no significant difference in toxicity between the latter compounds (*p* > 0.05). The present study showed that IFD seems to be a promising scolicidal agent and can be further tested to become a candidate for CE treatment.

## 1. Introduction

Hydatidosis (cystic echinococcosis, CE) is one of the most important helminthic diseases caused by *Echinococcus granulosus*, which is observed in the small intestine of dogs as the main host [1]. Humans and domestic livestock can be intermediary hosts; in their organs, including the liver, kidneys, lungs, and brain, hydatid cysts can develop [2]. CE remains a medical problem and represents a financial and public health concern in humans and animals in numerous parts of the world. CE is endemic in Northern Africa, the eastern part of the Mediterranean area, Central Asia, the Siberian region, Baltic areas, Southern and Eastern Europe, and other sheep-raising areas [2,3]. Currently, the therapeutic procedures for CE are quite limited. The existing approaches for the treatment of human CE include chemotherapy, surgery, and PAIR, the last consisting of puncture, aspiration, injection, and re-aspiration [2,4]. At present, several drugs, including the azoles derivatives, albendazole and mebendazole, are used in CE treatment [5]. Whenever cysts are few and small, these derivatives can be effective, but they can also produce harmful effects and sometimes fail in treatment protocols [6]. Up to now, surgery is still the preferred treatment for CE, especially when cysts are enormous and affect critical organs such as the heart or brain. During surgery, avoiding spillage of the cyst contents (protoscoleces) is essential to exclude the risk of immunological reactions, re-infection, and even death. Therefore, the utilization of scolicidal agents is critical to reduce the reappearance rate [2,4]. To date, natural medicines have been used for the treatment of several diseases, due to their relative harmlessness and good efficacy. The utilization of plants and spices has an extended history in the traditional medication of numerous diseases [7,8].

Sesquiterpenes are C15 organic, lipophilic compounds belonging to the group of terpenoids and are formed in the cytosol through the mevalonate pathway from the condensation of three isoprene units [9,10]. As volatile components of many plant essential oils, they are primarily used in medicines, as well as in cosmetics and perfumery.

**Figure 1:**
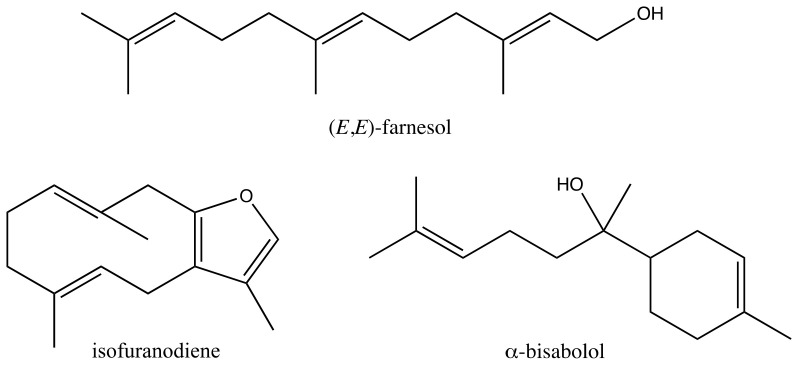
Chemical structures of the sesquiterpenes tested on *Echinococcus granulosus* protoscoleces.

Isofuranodiene (IFD), a furan-containing sesquiterpene (Figure 1), is the main volatile component of wild celery (*Smyrnium olusatrum* L.), a forgotten vegetable widely cultivated in the Mediterranean area until the Middle Ages and then abandoned after the domestication of common celery (*Apium graveolens* L.). IFD has been found to exert several bioactivities, such as anticancer, neuritogenic, anti-inflammatory, antimicrobial, antiprotozoal, insecticidal, and acaricidal [11,12,13,14,15,16,17,18,19,20,21]. IFD is solid at ambient temperature but may undergo thermal degradation (Cope rearrangement) with an increase of temperature [22,23].

α-Bisabolol (BSB) is a monocyclic sesquiterpene alcohol (Figure 1) occurring in the essential oil of chamomile (*Matricaria chamomilla* L.) and used in cosmeceutical and pharmaceutical applications due to its anti-inflammatory and skin-healing properties [24,25]. BSB is also an ingredient in several perfumes, soaps, detergents, and personal hygiene products [26]. Recently, BSB was shown to be effective against various tumors, such as pancreatic cancer [27], liver carcinoma, acute leukemia [28,29,30,31], and glioma [32].

Farnesol (FOH) is an acyclic sesquiterpene alcohol (Figure 1) formed from the dephosphorylation of farnesyl pyrophosphate (FPP), the key precursor of all the sesquiterpenes [33]. FOH was first isolated from *Vachellia farnesiana* (L.) Wight & Arn, also known as acacia farnese [34], and occurs in nature with four different isomers. At ordinary temperatures, FOH is a liquid oil with a sweet odor, and is thus employed in perfumery and cosmetic applications [35], as well as in the food industry as a flavoring agent [34]. As a component of plant essential oils, FOH is ubiquitous, being present in numerous herbs, such as lemon grass, pine, rose, chamomile, arnica, tuberose, wormwood, musk, citrus, neroli, and cyclamen [36,37]. FOH has been recognized to play a critical role in apoptosis, cell signaling, and proliferation [38,39,40]. This compound is endowed with anti-inflammatory and anticancer activities and is used for the palliation of allergic asthma, edema, and gliosis [41,42].

To the best of our knowledge, there are no reports dealing with the activity of IFD, BSB, and FOH on protoscolex of *E. granulosus*. Thus, in the present research, we studied the scolicidal activity of these three sesquiterpenes in order to find a promising natural scolicidal agent.

## 2. Results

Mortality rates of protoscoleces in IFD-, BSB-, FOH-, and albendazole-treated groups over different times of exposure were significantly different (*p* < 0.05). Significant effects of the treatment, (F4,260 = 2.877, *p* < 0.001), tested concentration (F7,304 = 310.56, *p* < 0.001), and time of exposure (F3,132 = 1.561, *p* < 0.001) were observed. The overall toxicity of IFD against *E. granulosus* protoscoleces differed significantly from those of FOH and BSB (*p* < 0.05). At the same time, there was not a significant difference between treatments with FOH and BSB (*p* > 0.05).

**Figure 2:**
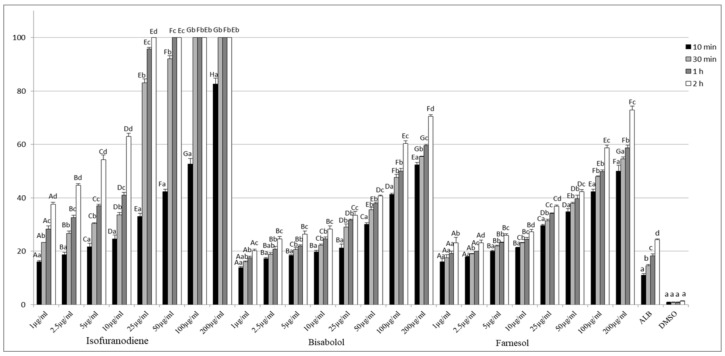
Mortality (%) of *E. granulosus* protoscoleces over different times of exposure to isofuranodiene, α-bisabolol, and farnesol. Within each concentration of tested compounds, columns marked with different letters (lowercase) are significantly different between times of exposure (Repeated measures ANOVA, Bonferroni test, *p* < 0.05). Within each tested compound, columns marked with different letters (uppercase) at each time of exposure (10 min, 30 min, 1 h, and 2 h, separately) are significantly different between concentrations (ANOVA, Tukey’s HSD test, *p* < 0.05).

As can be seen in Figure 2, IFD showed high scolicidal activity against *E. granulosus*. After 10 min of treatment, IFD, at 200 μg/mL, killed 82.66% of protoscoleces. At the same time point and the same concentration, BSB and FOH caused mortality rates of 52.33 and 50.06%, respectively. After 1 h, IFD at concentrations of 50, 100, and 200 µg/mL killed 100% of protoscoleces. On the other hand, at the same time point, FOH and BSB only led to mortality rates higher than 50% at a concentration of 200 µg/mL. After 1 h of exposure, IFD at all tested concentrations showed significantly higher toxicity in comparison to the other two treatments (*p* < 0.05).

After 2 h of treatment, all tested concentrations of IFD showed a statistically significant difference (*p* < 0.05) and caused considerable toxicity on *E. granulosus*, with mortality rates higher than 50%. At the same time point, 1 and 2.5 µg/mL of BSB and 2.5, 5, and 10 µg/mL of FOH did not show a statistically significant difference (*p* > 0.05).

In the control group, the mortality rate of protoscoleces after 2 h of treatment was 1.3%, while the mortality for albendazole as the positive control was 24.33%. Results also showed that the scolicidal activity of all compounds was significant (*p* < 0.05) compared to the control group at all exposure times. 

The LC_50_ and LC_90_ values of the tested compounds against protoscoleces of *E. granulosus* are shown in Table 1. IFD was the most effective sesquiterpene, with LC_50_ and LC_90_ values of 8.87 and 25.48 µg/mL, respectively. There was no significant difference between BSB (LC_50_ of 103.2 µg/mL) and FOH (LC_50_ of 113.68 µg/mL), due to an overlapping 95% confidence limit.

## 3. Discussion

From the commencement of humanity to the present, natural compounds have been used to treat numerous diseases [8]. Herbal remedies have been a popular form of alternative medicine worldwide, due to low toxicity, low price, high accessibility, and high efficiency [3,43]. 

IFD is an extremely hydrophobic molecule, which can be smoothly soaked up by the cell membrane, thus triggering perturbation of the phospholipid bilayer. Its greater efficacy against CE protoscoleces, compared to FOH and BSB, could be due to the presence of the furan ring, which increases the lipophilic character of IFD. Furthermore, the electron delocalization occurring on the furan ring turns its reactivity toward different functional groups of biological entities [13].

Regarding cytotoxicity, the work of Brunetti et al. [21] showed that IFD is relatively safe to normal human astrocytes (NHA), up to doses of 250 mM, whereas no LD_50_ values for animals have been reported so far. On the other hand, the acute toxicity of BSB and FOH after oral administration in rats is quite low, with LD_50_ values > 5 g/kg [26,35].

Up to now, various investigations have been done on the effects of several plant-derived compounds on *E. granulosus* protoscoleces. Tabari et al. [44] have reported the significant toxic effect of essential oils of *P. roseum* and *F. gummosa* on *E. granulosus* protoscoleces, with LC_50_ values of 8.52 and 17.18 μg/mL, respectively. Among the main constituents of these essential oils, β-pinene and citronellol were the most toxic, with LC_50_ values of 2.2 and 4.88 μg/mL, respectively [44]. Fabbri et al. [45] demonstrated that myrcene, a monoterpenoid compound and the main volatile constituent of *Cannabis sativa* L. (hemp) [46], was as effective as albendazole against germinal cells and protoscoleces of the murine cysts of *E. granulosus*. The scolicidal activity of the phenolic monoterpene, thymol, has been reported by Elissondo et al. [47]. They showed that thymol, at a concentration of 10 μg/mL, reduced the viability of protoscoleces to 53% after 12 days of incubation in culture media. Further studies supported the toxicity of thymol on protoscoleces by adding thymol (40 mg/kg) to albendazole (5 mg/kg) for the treatment of mice infected with *E. multilocularis*. The combination of these two compounds resulted in a higher antiparasitic effect, reduction of cyst weight, and severe damage to protoscoleces. High doses of thymol (250 μg/mL), after 2 min of exposure, resulted in about 90% mortality in protoscoleces under in vitro conditions. Based on the results of the study, thymol was suggested as a scolicidal in CE surgery [48]. Carvacrol, the main volatile compound of *Origanum vulgare* L. (oregano) and *Thymus vulgaris* L. (thyme) essential oils, showed in vitro and in vivo activity against *E. granulosus* protoscoleces at 10 μg/mL [49].

## 4. Materials and Methods

### 4.1. Isolation and Crystallization of Isofuranodiene

A total of 600 mL of *n*-hexane was added to 20 g of *S. olusatrum* flower essential oil, and the solution was stored at −20 °C for 5 days. The crude crystals which precipitated from the solution were filtered off and crystallized using hot methanol, thus achieving pure white needles of isofuranodiene (C_15_H_20_O, IFD, HPLC purity ~99%, yield = 79%). Accurate 1D (^1^H-NMR and ^13^C-NMR) NMR studies were carried out on a Bruker Avance III 500 MHz spectrometer (Bruker, Billerica, MA, USA), and the comparison with the data reported in the literature helped us to confirm IFD’s structure [18]. The chemical shift values are expressed in δ values (ppm), and coupling constants (*J*) are in hertz; tetramethylsilane (TMS) was used as an internal standard. Proton chemical data are reported as follows: chemical shift, multiplicity (s = singlet, d = doublet, dd = doublet of doublets, t = triplet, dt = doublet of triplets, q = quartet, m = multiplet, brs = broad singlet), coupling constant (s), and integration. No traces of curzerene were detected in this sample by ^1^H and ^13^C-NMR.

^1^H-NMR (DMSO-*d*_6_): *δ* 1.23 (s, 3H, CH_3_), 1.65 (s, 3H, CH_3_), 1.72–1.85 (m, 1H, H-1a), 1.89 (s, 3H, CH_3_), 2.08 (dt, *J* = 2.6, 8.1 Hz, 2H, H-2), 2.25 (d, *J* = 11.8 Hz, 1H, H-1b), 2.97–3.11 (m, 2H, H-7), 3.42 (q, *J* = 16.8 Hz, 2H, H-10), 4.67 (d, *J* = 10.1 Hz, 1H, H-6), 4.95 (t, *J* = 8.1 Hz, 1H, H-4), 7.23 (s, 1H, H-12). ^13^C-NMR (DMSO-*d*_6_): 8.9 (C-16), 16.2 (C-14), 16.5 (C-15), 24.3 (C-7), 27.3 (C-10), 39.5 (C-1), 40.9 (C-2), 119.1 (C-8), 121.7 (C-3), 121.9 (C-11), 127.8 (C-4), 128.9 (C-6), 134.5 (C-5), 136.1 (C-12), 149.9 (C-9). MS (API-ESI): *m/z* 217.15 [M + H]^+^. Anal. calcd. for (C_15_H_20_O) C, 83.28; H, 9.32; found: C, 83.26; H, 9.33.

The purity of IFD (~99%) was assessed by HPLC using an HP-1100 series (Agilent Technologies, Palo Alto, CA) LC system equipped with a diode array detector (DAD). The separation was accomplished on a Kinetex^©^ PFP (100A, 100 × 4.6 mm i.d., 2.6 mm) thermostatted at 40 °C, using H_2_O (Milli-Q SP Reagent Water System, Millipore, Bedford, MA) and CH_3_CN (Sigma-Aldrich, Milan, Italy, 99.8%) as mobile phases A and B, respectively. The gradient elution (1.0 mL/min) was set as follows: 0–15 min (40% B), 15–30 min (60% B). ISD was diluted in CH_3_CN and injected (5 mL) into HPLC using disposable Minisart SRP4 filters, with a pore width of 0.45 mm (Chromafil PET-20/25, Sartorius Stedim Biotech GmbH, Goettingen, Germany). The peak of IFD eluted at a retention time of 22.432 min was monitored at different wavelengths (220, 230, and 254 nm).

α-Bisabolol, BSB (CAS number 23089-26-1) and farnesol, FOH (CAS number: 4602-84-0, as a mixture of isomers) were purchased from Sigma-Aldrich (Darmstadt, Germany).

### 4.2. Collection of Protoscoleces

Protoscoleces were recovered from the liver of a sheep with hydatidosis, which was slaughtered in Sari, Mazandaran Province, Iran, and was transferred to the parasitology lab of the Faculty of Veterinary Medicine, Azad University of Babol, Iran. Under sterile conditions, the contents of cysts containing fluids and protoscoleces were drained into a sterile flask, and the protoscoleces were allowed to settle down for 30 min. Next, the protoscoleces were collected and washed twice with a PBS (pH 7.2) solution. Finally, the number of protoscoleces per mL was adjusted to 2 × 10^3^ in a 0.9% NaCl solution, with more than 90% viability, as determined by eosin-exclusion test.

### 4.3. Protoscoleces Viability Test

To define the ratio of viable protoscoleces, the fluid, including protoscoleces, was decanted onto the slide, and eosin solution 0.1% (1 g of eosin powder in 1000 mL of distilled water) was sited close to the sample with an identical capacity. Finally, protoscoleces and the stain were mixed slowly, and the protoscoleces were analyzed after 10 min under a light microscope by counting live protoscoleces (which do not absorb color) and dead ones (eosin enters the cell and protoscoleces become red).

### 4.4. In Vitro Scolicidal Activity 

IFD, BSB, and FOH at 8 concentrations in 10% DMSO, corresponding to 1, 2, 5, 10, 25, 50, 100, and 200 µg/mL, were added to test tubes containing 1000 protoscoleces and mixed gently. Albendazole at a concentration of 2 µg/mL was used as the control. Tubes were kept for 10, 30, 60, and 120 min at 37 °C. After these times, the supernatant was detached, and protoscoleces were mixed with 50 µL of 0.1% eosin stain (Sigma-Aldrich, St. Louis, MO, USA). After 10 min, the protoscoleces were smeared on a slide, and all surfaces of the slide were checked under a light microscope. The number of dead protoscoleces was counted, and mortality rates were recorded [50].

### 4.5. Statistical Analysis

Data analyses were done using the SPSS statistical package (version 23.0) (SPSS Inc., Chicago, IL, USA). Differences between the mean of the mortality rate of each concentration of tested compounds at different exposure times were analyzed by one-way analysis of variance (ANOVA) and Tukey-HSD as post hoc tests. The repeated measures were performed to analyze different time points of exposures. For calculation of 50% lethal concentration (LC_50_) and 90% lethal concentration (LC_90_) of IFD, BSB, and FOH, a Probit regression analysis was used. For all analyses, *p* < 0.05 was considered statistically significant.

## 5. Conclusions

There are many reports on the toxicity of terpenes against veterinary and medically important parasites, but most of them focused on monoterpenoid compounds [25,51,52]. On the other hand, studies on sesquiterpenes are limited in the literature. The present study demonstrated, for the first time, a promising in vitro scolicidal activity for IFD. This may represent a step forward in the search for new antiparasitic agents, at a time when there is an urgent need for novel scolicidals. This promising result can be confirmed by further studies on the in vivo scolicidal activity of IFD for a better understanding of its activity and potential side effects in animals and human beings during CE surgery. 

## Figures and Tables

**Table 1:** Lethal concentrations (LC_50_ and LC_90_ values) of isofuranodiene, bisabolol, and farnesol against *Echinococcus* *granulosus* protoscoleces.

Tested Compound	Concentration (µg/mL)	1 h Mortality (%) ± SE ^a^	LC_50_ (µg/mL) (LCL-UCL)	LC_90_ (µg/mL) (LCL-UCL)	χ^2^ (df) ^b^
**Isofuranodiene**	1	28.33 ± 1.20	8.87 (5.76–11.87)	25.48 (19.75–31.96)	9.846 (4) n.s.
	2.5	32.66 ± 0.88			
	5	37.00 ± 0.85			
	10	41.00 ± 1.10			
	25	95.66 ± 0.61			
	50	100.00 ± 0.00			
	100	100.00 ± 0.00			
	200	100.00 ± 0.00			
**α** **-Bisabolol**	1	17.60 ± 0.53	103.20 (84.62–127.16)	341.47 (288.1–404.86)	2.130 (5) n.s.
	2.5	20.74 ± 1.14			
	5	22.00 ± 0.47			
	10	24.55 ± 0.76			
	25	31.66 ± 0.46			
	50	37.88 ± 0.41			
	100	50.06 ± 1.02			
	200	59.55 ± 0.46			
**Farnesol**	1	19.11 ± 0.90	113.68 (97.89–168.31)	386.78 (311.98–451.59)	7.494 (5) n.s.
	2.5	20.02 ± 0.02			
	5	23.29 ± 0.20			
	10	24.49 ± 0.69			
	25	34.21 ± 0.12			
	50	39.66 ± 1.33			
	100	49.89 ± 0.54			
	200	58.65 ± 1.20			

SE—standard error, LCL 95%—lower confidence limit, UCL 95%—upper confidence limit, n.s.—not significant (*p* > 0.05). ^a^ values are mean ± SE of three replicates. ^b^ Chi-square, df—degrees of freedom.
